# A before and after study of the impact on obstetric and perinatal outcomes following the introduction of an educational package of fetal heart rate monitoring education coupled with umbilical artery lactate sampling in a low resource setting labor ward in South Africa

**DOI:** 10.1186/s12884-019-2552-8

**Published:** 2019-11-06

**Authors:** Emma R. Allanson, Robert C. Pattinson, Elizabeth A. Nathan, Jan E. Dickinson

**Affiliations:** 1 Division of Obstetrics and Gynaecology, Faculty of Health and Medical Sciences M550, The University of Western Australia, 35 Stirling Hwy, Crawley, WA 6009 Australia; 2 SAMRC/UP Maternal and Infant Health Care Strategies Unit, Department of Obstetrics and Gynaecology, University of Pretoria, Unit Private Bag X323 Arcadia, Pretoria, 0007 South Africa

**Keywords:** Lactate, Caesarean, South Africa, Maternal, Neonatal

## Abstract

**Introduction:**

Rates of cesarean section (CS) are increasing and abnormal fetal heart rate tracing and concern about consequent acidosis remain one of the most common indications for primary CS. Umbilical artery (UA) lactate sampling provides clinicians with point of care feedback on CTG interpretation and intrapartum care and may result in altered future practice.

**Materials and methods:**

From 3rd March - 12th November 2014 we undertook a before and after study in Pretoria, South Africa, to determine the impact of introducing a clinical package of fetal heart rate monitoring education and prompt feedback with UA cord lactate sampling, using a hand-held meter, on maternal and perinatal outcomes.

**Results:**

Nine hundred thirty-six consecutive samples were analyzed (pre *n* = 374 and post *n* = 562). There was no difference in mean lactate (4.6 mmol/L [95%CI 4.4–4.8] compared with 4.9 mmol/L [95%CI 4.7–5.1], *p* = 0.089). Suspected fetal compromise was reduced in the post-intervention period: 30·2% vs 22·1%, aOR 0·71, 95% CI 0·52–0·96, *p* = 0·027. Cesarean section rates were significantly reduced in the univariate analysis: pre- 40·3% vs post-intervention 31·6% (*p* = 0·007). This reduction remained significant when adjusted for previous cesarean section, primiparity, maternal HIV infection and preterm birth (aOR 0·72, 95%CI 0·54–0·98, *p* = 0·035). Neonatal outcomes did not differ between the two groups.

**Conclusion:**

The introduction of a clinical practice package of fetal heart rate monitoring education combined with routine UA cord lactate sampling has the potential to reduce the cesarean section rate without increasing adverse neonatal outcomes in a low-resource setting.

## Key message

Both rising cesarean section rates and high perinatal mortality are issues of global concern. We demonstrate using a simple method of testing umbilical artery lactate at birth, that it is possible to reduce cesarean section rates without worsening neonatal morbidity in a low resource setting.

## Introduction

The rates of cesarean section (CS) are increasing globally [1], although both the total rates and the rate of rise vary considerably between low, middle, and high income countries [2]. While there is little agreement about the optimal rate of CS, there is some evidence that increasing rates above a certain threshold may not result in a corresponding improvement in maternal and perinatal mortality [3]. Moreover, CS, while a life-saving procedure that all women must have access to when required, is also associated with potential adverse maternal outcomes in both the index and future pregnancies [4]. Balancing the indication for CS with its potential risks is a critical clinical and public health challenge.

Abnormal fetal heart rate tracing or suspected fetal distress with concern for consequent hypoxia and acidemia remains one of the most common indications for the first CS (with rates cited between 10 and 32%) [5–8]. However, the rates of pathological acidemia in neonates generally is far lower than this level, and although there are documented benefits, the use of electronic fetal heart rate monitoring in labour has not been shown to improve neonatal mortality [9]. It is important to consider fetal physiology in the intrapartum period and cardiotocograph (CTG) interpretation in great detail given the need to balance the role that intrapartum hypoxia plays in global perinatal deaths, with the role that potentially unnecessary CS play in maternal morbidity and mortality.

Objective measurements of intrapartum fetal hypoxia (such as pH or lactate sampled from the fetal scalp) may improve upon the known limited specificity of CTG and reduce the rate of unnecessary cesarean sections [10, 11]. The cost of the equipment to do this, alongside high rates of maternal HIV infection makes this prohibitive in some units around the world. Universal umbilical cord-gas sampling after delivery has been suggested as a method to improve the specificity of the CTG [12]. Paired cord arterial and venous gas sampling is both time and resource intensive however, particularly outside of high-income country settings. To that end, it is possible to introduce inexpensive bedside point of care umbilical artery (UA) lactate testing, which provides clinicians feedback on intrapartum care and potentially alters practice for future cases, which may partly mitigate the poor specificity of the CTG. Therefore, we conducted a before and after study in a tertiary labor ward in Pretoria, South Africa, to determine the impact of introducing UA lactate sampling, in conjunction with education on CTG interpretation and fetal and lactate physiology, on maternal and perinatal outcomes.

## Materials and methods

Kalafong Hospital is a tertiary hospital in Pretoria, South Africa, affiliated with the University of Pretoria. In 2014, there were 6499 births in the unit. During the period 3rd March to the 12th November 2014 we conducted a before and after study to determine the impact of umbilical artery lactate sampling, combined with an educational program on fetal heart rate monitoring, on maternal and neonatal outcomes.

The study groups included prospectively collected samples of umbilical artery lactate obtained by the primary researcher (EA), or a doctor in the unit trained by the primary researcher. We have presented the specifics of UA sampling in the unit, and the data points collected previously [13]. Briefly, after maternal written consent was obtained, the umbilical cord was clamped and cut within 1 minute of birth. A small arterial blood sample (< 0.5uL) was then taken from a double clamped segment of the remaining umbilical cord, prior to delivery of the placenta. The lactate level was measured (mmol/L) on a Roche Accutrend PlusTM© hand-held lactate meter (Rotkreuz, Switzerland). The coefficient of variation (CV) for this lactate meter is 1.8–3% [14]. All women aiming to have a vaginal birth were eligible to be consented for the trial, regardless of their ultimate birth mode.

During the period 3rd March to 18th of July, 2014, a prospective cohort of women were consented to be enrolled in the study. As this was a blinded cohort, this was a convenience sample, obtained within the capacity of a single researcher (EA). The results of the lactate sampling in this cohort were blinded to the clinicians (midwives, registrars, and consultants) managing the intrapartum care of women. This cohort served as the baseline for the unit, the results of which have been published previously [13]. Before the completion of the cohort, all midwives and registrars in the unit underwent a training course in fetal physiology, lactate physiology and cardiotocograph (CTG) interpretation, conducted by the primary researcher (EA). The course consisted of a pre-test, a series of didactic lectures, interactive application of knowledge with CTG examples, and a post-test. Subsequently, from 21st July to 12th November 2014, lactate sampling continued, with the results made immediately available to the clinicians managing the intrapartum care of women in the labor ward. Furthermore, all lactate samples obtained in the previous 24 h were reviewed at the daily morning labour ward meetings of all medical staff (interns, registrars, and consultants), and twice weekly, an in-depth review of interesting or challenging cases was facilitated by the primary researcher (EA). Daily reviews involved correlating CTG interpretation pre-birth with neonatal outcomes, including the UA lactate. During the two study periods, basic data on all births (regardless of recruitment to the study) were collected, including number of births, mode of birth, and admissions to the neonatal nursery (all levels).

### Statistics and analysis

The estimation of sample size was performed using standardised normal lactate mean and standard deviation. A sample size of 265 in each of the pre and post intervention groups would achieve an 80% power to detect a difference of 0.25 standard deviations (approximately 8% difference in means) in lactate measurements between groups using a two-sided sample t-test at 5% significance level. This sample size would also achieve at least 80% power to detect an R-squared of 0.05 attributed to one or more independent variable/s while simultaneously adjusting for multiple other relevant covariates with an R-squared of 0.2 in a multiple linear regression analysis. Assuming a 15% attrition rate, the sample size was increased to 305 in each group for a total sample size of 610. ([Power and Sample Size (PASS 2008)].

To assess the lactate results, data were transformed to the natural logarithm to correct normality and summarised as geometric means and 95% confidence intervals (CI). Linear regression analysis was conducted on the log transformed lactate measurements and presented as estimated mean effects and 95% CI’s. A multivariate analysis on lactate levels, adjusted for primiparity, HIV, fetal problems in pregnancy and preterm birth, was done with lactate measurements log transformed for the analysis. Summary values of estimated mean effects and 95% confidence intervals were back transformed. Outcomes were summarised using means and standard deviations or medians, interquartile ranges and ranges for continuous data and frequency distributions for categorical data. Based on our previously published cohort study, a cut-off for an abnormal lactate was assumed to be 5·45 mmol/L [13]. Univariate comparisons were made using independent t-tests for continuous outcomes and Chi-square or Fisher exact tests for categorical comparisons. Multivariable logistic regression analysis was performed on maternal and neonatal outcomes including fetal distress, cesarean section, resuscitation, Apgar scores and admission to special care nursery. Results were summarised with unadjusted (OR) and adjusted odds ratios (aOR) and 95% CI’s.

Analyses of outcomes were adjusted for preterm birth, primiparity, HIV, previous uterine surgery or maternal problems in pregnancy (maternal outcome), and fetal problems during pregnancy (neonatal outcomes). Maternal problems in pregnancy included being an adolescent, having advanced maternal age (> 35), anemia, cervical incompetence, eclampsia, gestational diabetes mellitus, HELLP syndrome, hypertension without pre-eclampsia,, maternal medical conditions, no antenatal care, pre-eclampsia, preterm labour, syphilis, thyrotoxicosis, tuberculosis, urinary tract infection, uterine anomaly, or venous thromboembolic event. A pre-planned subgroup analysis was conducted on infants with birth weight < 2500 g compared with ≥2500 g and neonatal outcomes were assessed. A Bonferroni adjustment was applied such that the significance level for each comparison of pre- and post-intervention groups within birth weight strata was set to 0·025. All tests were two-sided and a *p*-value < 0·05 was considered statistically significant for the overall analysis. SPSS statistical software was used in data analysis (version 22·0, Armonk, NY: IBM Corp).

A protocol was developed for this study; it is not published but available upon request.

### Ethics

This study was approved by the ethics committees of The University of Western Australia Human Research Ethics Committee on the 26/02/2014 (Reference number RA/4/1/6581), the University of Pretoria, Pretoria, South Africa on the 10/02/2014 (Reference number 7/2014).

## Results

Three hundred ninety-seven samples were collected in the pre-intervention period from 2196 planned vaginal births and 2436 total births. Five hundred ninety-seven samples were collected in the post-intervention period from 1978 planned vaginal births and 2232 total births. After exclusion of 54 twin pregnancies, a total of 936 samples were analysed (pre *n* = 374 and post *n* = 562).

### Maternal characteristics

Maternal characteristics did not differ between groups (Table 1). The maternal HIV prevalence in the pre-intervention group was 21·8% (*n* = 79), with antiretroviral therapy (both prophylactic antiretroviral therapy and highly active antiretroviral treatment) used in 96·2%·of women antenatally. The maternal HIV prevalence in the post-intervention group was 23% (*n* = 123), with antiretroviral therapy in 92·4% of women. A comparison of the maternal and neonatal outcomes between the HIV and non-HIV infected mothers has been published elsewhere [15].

**Table 1:** Maternal characteristics in pre- and post-intervention groups

	Pre-intervention (*N* = 374)	Post –intervention (*N* = 562)	*p*-value
Maternal age (y)	27·4 (±6·1)	27·4 (±6·6)	0·897
Gestation (w)	38·8 (±3·1) (*n* = 345)	38·8 (±3·2) (*n* = 504)	0·963
Gravidity	2 (1–3;1–7)	2) 1–3;1–8)	0·672
Parity	1 (0–2;0–5)	1 (0–2;0–7)	0·820
Nulliparous	136 (36·7%)	195 (36·2%)	0·899
Previous uterine surgery	53 (14·7%)	56 (10·6%)	0·072
HIV status	79 (21·8%)	123 (23·0%)	0·655
HIV treatment	75 (96·2%)	109 (92·4%)	0·369

Data summaries are mean (±sd), median (IQR,R) or N (%), as appropriate

### Pregnancy and labour

The pre-intervention group had a higher rate of maternal-related pregnancy complications (41·8% vs 22·4%, *p* < 0·001) and were also more likely to have intrapartum complications (55·6% vs 45·9%, *p* = 0·004), fetal distress (30·2% vs 22·1%, *p* = 0·005), other labor complications (3·5% vs 1·4%, *p* = 0·038) and be transferred intrapartum from a referral unit (19·5% vs 7·3%, *p* < 0·001). The intrapartum complications are outlined in Table 2. Other labor complications included were infrequent in both groups and included twin pregnancy diagnosed intrapartum, the development of isolated intrapartum hypertension, acute twin to twin transfusion syndrome and single unusual occurrences such as “patient found on antenatal ward with fetal head delivered”, “patient pushing from four centimetres”, maternal collapse at full dilatation, and failed instrumental delivery at local clinic prior to transfer.

**Table 2:** Intrapartum complications in pre- and post-intervention groups

	Pre-intervention *N* = 374 N (%)	Post–intervention *N* = 562 N (%)	*p*-value
Any	208 (55·6%)	258 (45·9%)	0·004
Abruption	7 (1·9%)	7 (1·2%)	0·440
Chorioamnionitis	0	4 (0·7%)	0·155
Delayed progress 1st stage	40 (10·7%)	85 (15·1%)	0·051
Delayed progress 2nd stage	15 (4·0%)	23 (4·1%)	0·950
Intrapartum hemorrhage	7 (1·9%)	12 (2·1%)	0·779
Malpresentation	8 (2·1%)	14 (2·5%)	0·728
Meconium stained amniotic fluid	27 (7·2%)	40 (7·1%)	0·953
Augmentation of labor	13 (3·5%)	25 (4·4%)	0·460
Shoulder dystocia	9 (2·4%)	7 (1·2%)	0·180
Fetal distress	113 (30·2%)	124 (22·1%)	0·005
Other^a^	13 (3·5%)	8 (1·4%)	0·038
Intrapartum transfer	73 (19·5%)	41 (7·3%)	< 0·001

^a^Other = see note in main body of text

### Lactate results

A lactate result was recorded for 351 (94%) of women recruited in the pre-intervention group and 542 (96%) of women recruited post-intervention. There was no difference in the mean lactate result (4.6 mmol/L [95%CI 4.4–4.8] compared with 4.9 mmol/L [95%CI 4.7–5.1], *p* = 0.089) or in babies born with a lactate greater than 5.45 mmol/L (122 (34.8%) compared with 210 (38.7%), *p* = 0.228). There was no difference in mean lactate levels between the two groups when adjusted for primiparity, HIV infection, fetal problems in pregnancy and preterm birth (unadjusted OR 1.05 [95%CI 0.99–1.13], compared with an adjusted OR 1.06 [95%CI 0.99–1.13], *p* = 0.108).

### Birth characteristics

Pre-intervention women were more likely to have a cesarean section due to suspected fetal distress (21·4% vs14·9%, *p* = 0.011) and vaginal birth after cesarean (VBAC) in labour (8·0% vs 2·5%, *p* < 0·001). Other indications for cesarean delivery were similar between the two groups. Suspected fetal distress during labor was reduced in the post-intervention period: pre- 30·2% vs post-intervention 22·1%, aOR 0·71, 95% CI 0·52–0·96, *p* = 0·027. Cesarean section rates were significantly reduced in the univariate analysis: pre- 40·3% vs post-intervention 31·6% (*p* = 0·007). This reduction remained significant when adjusted for previous cesarean section, primiparity, maternal HIV infection and preterm birth (aOR 0·72, 95%CI 0·54–0·98, *p* = 0·035).

### Neonatal characteristics

The neonatal characteristics in the two groups are outlined in Table 3. There was no difference in neonatal outcomes when adjusted for primiparity, HIV infection, fetal problems in pregnancy and preterm birth. When stratified by birth weight (< 2500 g and ≥ 2500 g), there was no difference in any outcomes, including lactate results (see Table 4).

**Table 3:** Neonatal characteristics pre- and post-intervention groups

	N*	Pre-intervention	N*	Post–intervention	*p*-value
Preterm (<37w)	345	64 (18·6%)	504	99 (19·6%)	0·691
Birthweight (g)	363	3033·9 (±623·5)	520	3006·4 (±641·2)	0·527
Lactate > 5·45		122 (34·8%)		210 (38·7%)	0·228
Resuscitation	344	71 (20·6%)	532	129 (24·2%)	0·214
Apgar < 7 at 1 min	365	45 (12·3%)	526	83 (15·8%)	0·149
Apgar < 7 at 5 min	366	20 (5·5%)	526	35 (6·7%)	0·468
Special care nursery admission	374	56 (15·0%)	562	80 (14·2%)	0·754
Admission type	367		558		
None		318 (86·6%)		480 (86·0%)	
High dependency unit		8 (2·2%)		22 (3·9%)	0·387
Intensive care unit		14 (3·8%)		15 (2·7%)	
Neonatal ward		27 (7·4%)		41 (7·3%)	

Data summaries are mean (±sd) or N (%), as appropriate

* N reflects cases for which data were available for the variable

**Table 4:** Neonatal outcomes stratified by birth weight < 2500 g and ≥ 2500 g

	Birth weight < 2500 g			Birth weight ≥ 2500 g		
	Pre-intervention	Post –intervention	*p*-value	Pre-intervention	Post-intervention	*p*-value
	*n* = 52	*n* = 78		*n* = 311	*n* = 442	
Birth weight (g)	1937.48 (±556.1)	1836.35 (±534.2)	0.300	3217.2 (±409.6)	3212.9 (±386.3)	0.884
	*n* = 46	*n* = 68		*n* = 291	*n* = 417	
Gestation (w)	35.4 (±4.7)	34.6 (±4.4)	0.372	39.3 (±2.4)	39.5 (±2.3)	0.454
Preterm (<37w)	25 (54.3%)	47 (69.1%)	0.109	38 (13.1%)	47 (11.3%)	0.472
	*N* = 52	*N* = 75				
Lactate^a^	4.4 (3.8–5.2)	4.9 (4.4–5.4)	0.263	4.7 (4.4–4.9)	4.8 (4.6–5.1)	0.333
Lactate > 5.45	19 (36.5%)	27 (36.0%)	0.950	99 (34.3%)	165 (38.8%)	0.215
Resuscitation	22 (43.1%)	34 (44.2%)	0.909	47 (16.5%)	90 (20.5%)	0.181
Apgar< 7 at 1 min	14 (27.5%)	24 (32.0%)	0.585	30 (9.8%)	56 (12.8%)	0.209
Apgar < 7 at 5 min	7 (13.7%)	16 (21.3%)	0.278	12 (3.9%)	19 (4.3%)	0.771
Special care nursery admission	25 (48.1%)	41 (52.6%)	0.616	30 (9.6%)	38 (8.6%)	0.621
Admission type						
None	27 (55.1%)	37 (48.7%)	0.687	281 (91.5%)	402 (91.2%)	0.664
Ward	15 (30.6%)	24 (31.6%)		6 (2.0%)	13 (2.9%)	
High dependency unit / Intensive care unit	7 (14.3%)	15 (19.7%)		20 (6.5%)	26 (5.9%)	

Data summaries are mean (±sd) or N (%), as appropriate

*P*-values less than 0.025 considered statistically significant with a Bonferroni correction

^a^Data are summarised as geometric mean and 95% confidence intervals

### Mode of delivery and neonatal outcomes for all deliveries

There were 2436 births during the entire pre-intervention period, and 2232 during the post-intervention period. During the pre-intervention period there were 932/2436 cesarean deliveries (38·3%), which decreased in the post-intervention period to 653/2232 (29·3%, *P* < 0·001). There was no change in rate of elective cesarean sections; 240/2436 (9.9%) to 254/2232 (11.4%, *P* = 0.09). There was no difference in instrumental births 73/2436 (3%) to 81/2232 (3.6%, *P* = 0.227). Emergency cesarean deliveries decreased from 692/2436 (28·4%) to 399/2232 (17·0%, *P* < 0·001), and neonatal admissions decreased from 450/2436 (18·5%) to 349/2232 (15·6%, *P* = 0·010). A diagnosis was recorded for 360 (80%) admissions in the pre-intervention period and 278 (80%) of admissions in the post-operative period. The commonest reasons for admission in the pre-intervention period were respiratory distress syndrome (RDS) (19.7%), neonatal jaundice (NNJ) (13.1%), congenital infections (8.6%), hypoglycemia (7.5%), hypoxic ischemic encephalopathy (HIE) (7.2%), and prematurity (7.2%). In the post intervention period the commonest reasons were RDS (19.1%), NNJ (12.2%), hyaline membrane disease (HMD) (10.1%), transient tachypnea of the newborn (TTN) (10.1%), low birth weight (LBW) (8.6%) and HIE (7.9%).

## Discussion

This study explored the impact of an educational program coupled with the introduction of UA lactate sampling in a low-resource setting on intrapartum outcomes. Previous studies have been based in high-income settings [16] and it was not clear whether the previously observed benefits in earlier studies would translate to a lower resource centre. While there was no significant difference in the cord lactate results between the two groups, we found a reduction in the CS rate without worsening any of neonatal outcomes. The largest contributor to this reduction was in CS for suspected fetal compromise. A significant reduction post-intervention was observed in CS, emergency CS, and neonatal admissions for all women regardless of whether they were recruited to the study for lactate measurement or not. While these overall results are not adjusted for confounding factors, they are reassuring; the falling CS rate has not worsened fetal and neonatal outcomes, and, based on the reduction in overall neonatal unit admission rate, may have improved them. The impact of an educational program on fetal physiology and intrapartum heart rate monitoring demonstrated benefits that were translated to all women in the labour ward and we speculate this should be sustained when coupled with the introduction of universal cord lactate sampling for ongoing clinician outcome feedback.

There is evidence that the lack of appropriate interpretation of fetal monitoring in South Africa contributes to the unacceptably high intrapartum and early neonatal mortality rates [17, 18]. Equally, there is evidence that the process of quality of care audit and the feedback to clinicians of the UA lactate results may improve maternal and neonatal outcomes [12, 19]. Given the reduction in suspected fetal compromise without an increase in adverse neonatal outcomes that occurred following our intervention, we hypothesise that the objective feedback available to clinicians from the UA lactate result highlighted recognition on CTG interpretation of the “true” acidemic cases. This theory may be supported by the trend to less neonatal nursery admissions in the study group and the significantly less admissions overall in the post-intervention study period, regardless of trial recruitment status. In addition, we observed an increase in delayed labour progress in the post-intervention group without worse neonatal outcomes; it may be that less early interventions for suspected fetal distress allowed other potential complications of longer labours labor to become apparent.

Interestingly, there were less maternal problems in the post intervention period, which plausibly could have resulted in fewer fetuses at risk of compromise. There were, however, significantly less intrapartum transfers from referral facilities in the post-intervention period. The nature of an intrapartum transfer indicates the women are more likely to have maternal related pregnancy problems, necessitating up-referral to a tertiary unit [20, 21], and this may have accounted for some of the reduction in maternal complications in the post-intervention period.

There was no significant change in the elective CS rate, with the reduction in our study almost exclusively intrapartum non-elective CS, which remained significant when adjusted for primiparity. This is where we believe the strength of this intervention lies – in preventing the first CS, particular considering evidence such as that of Villar et al*,* demonstrating that intrapartum cesarean section in a sample of nearly 100,000 deliveries in 8 Latin American countries was associated with an increased risk in several maternal and neonatal morbidity and mortality [22]. The Robson classification for cesarean section is widely used to monitor CS rates internationally [23], with a particular focus on reducing cesarean section in nulliparous women (group 1 and 2 in the Robson classification) and those with a previous cesarean section, (group 5), with the latter a clear consequence of the former [1]. Although heterogeneous in the quality of included studies, a recent meta-analysis has demonstrated that quality of care audit using the Robson classification system to monitor cesarean section rates can result in a reduction in CS [24]. In our study we introduced a quality of care audit process and provided instant feedback to the clinicians in the unit, with a reduction in CS in women in groups 1 and 2 of the Robson classification.

Reduction in CS is of particular importance in South Africa, as women in this country are more likely to die following a CS compared with a vaginal birth [25]. This is particularly concerning as 22·6% of deliveries in South Africa are by CS, and in provincial tertiary hospitals, as is the unit in this study, the rate is 35·2% [26]. Moreover, maternal deaths related to CS have been increasing, and disturbingly, large numbers of these are due to perioperative haemorrhage [27], with surgical trauma being one of, if not the most common, reason for death [25, 28]. The reduction in CS in our study (both in the included cases and overall during the study period) has the potential for immediate and downstream effects for future pregnancies, as well as long-term outcomes for women and their families.

Finally, testing for potential fetal acidemia with lactate on a handheld, point of care device has several advantages. Firstly; it may be more feasible (both economically and in ease of use) than other measures (such as paired arterial and venous cord gas sampling), especially in the limited resource setting. Secondly, the hand-held device correlates well with laboratory analysers, adding validity to the process of point of care testing [29, 30].

### Strengths

To our knowledge this is the largest assessment of an intervention using umbilical lactate measurement and its impact in a low or middle income country on intrapartum obstetric management [16], and is likely representative of the unit as a whole. The presence of a single researcher on the labor ward (EA) to oversee the training of health care providers in sample collection and ensure complete data collection throughout the study period adds to the internal validity of the results.

### Limitations

It is difficult to separate the impact of the fetal physiology and CTG interpretation training with the lactate sampling on the change in practice following the intervention and indeed it is likely the combination of both that has created a management package. All staff who cared for women in our study had previously completed the Essential Steps in the Management of Obstetric Emergencies (ESMOE) course, which includes a module on CTG interpretation. This suggests that the addition of the UA lactate and the quality of care audit process around this was the main factor in practice change. However, ongoing revision of CTG interpretation is an essential element in labour ward practice and we recommend regular case review and education programs.

As part of this study, we introduced point of care testing, and although training was undertaken for all involved in the collection, we do not have paired samples (both arterial and venous) to provide confirmation of arterial sampling. We are, however, reassured by the fact that venous lactate is shown to be predictive of arterial lactic acidemia, and that the previous optimal cut-off for lactate we obtained using these samples was very similar to a large cohort including paired samples [13].

## Conclusion

The introduction of UA lactate has the ability to significantly reduce the CS rate without increasing adverse neonatal outcomes in a low-resource setting. Further research to expand the body of evidence on the use of UA lactate sampling in low and middle-income countries is needed.

## Data Availability

The datasets used and/or analysed during the current study are available from the corresponding author on reasonable request.

## Abbreviations

aOR: Adjusted odds ratio
AVD: Assisted vaginal delivery
CI: Confidence intervals
CS: Cesarean sections
CTG: Cardiotocograph
CV: Coefficient of variation
HIE: Hypoxic ischaemic encephalopathy
HIV: Human immunodeficiency virus
HMD: Hyaline membrane disease
LBW: Low birth weight
NNJ: Neonatal jaundice
OR: Odds ratio
RDS: Respiratory distress syndrome
TTN: Transient tachypnoea of the newborn
UA: Umbilical artery
VBAC: Vaginal birth after cesarean
